# The impact of *KIR/HLA* genes on the risk of developing multibacillary leprosy

**DOI:** 10.1371/journal.pntd.0007696

**Published:** 2019-09-16

**Authors:** Hugo Vicentin Alves, Amarilis Giaretta de Moraes, Afonso Carrasco Pepineli, Bruna Tiaki Tiyo, Quirino Alves de Lima Neto, Thais da Silva Santos, Jorge Juarez Vieira Teixeira, Eliane P. Ambrosio-Albuquerque, Ana Maria Sell, Jeane Eliete Laguila Visentainer

**Affiliations:** 1 Laboratory of Immunogenetics, Department of Basic Health Sciences, Maringa State University (UEM), Parana, Brazil; 2 Post Graduation Program in Biosciences and Physiopathology, Department of Clinical Analysis and Biomedicine, Maringa State University (UEM), Parana, Brazil; 3 Department of Biotechnology, Cell Biology and Genetics, Maringa State University (UEM), Parana, Brazil; Mahidol University, THAILAND

## Abstract

**Background:**

Killer-cell immunoglobulin-like receptors (KIRs) are a group of regulatory molecules able to activate or inhibit natural killer cells upon interaction with human leukocyte antigen (HLA) class I molecules. Combinations of KIR and HLA may contribute to the occurrence of different immunological and clinical responses to infectious diseases. Leprosy is a chronic neglected disease, both disabling and disfiguring, caused mainly by *Mycobacterium leprae*. In this case–control study, we examined the influence of KIRs and HLA ligands on the development of multibacillary leprosy.

**Methodology/Principal findings:**

Genotyping of *KIR* and *HLA* genes was performed in 264 multibacillary leprosy patients and 518 healthy unrelated controls (238 healthy household contacts and 280 healthy subjects). These are unprecedented results in which *KIR2DL2/KIR2DL2/C1/C2* and *KIR2DL3/2DL3/C1/C1* indicated a risk for developing lepromatous and borderline leprosy, respectively. Concerning to *3DL2/A3/A11+*, our study demonstrated that independent of control group (contacts or healthy subjects), this KIR receptor and its ligand act as a risk factor for the borderline clinical form.

**Conclusions/Significance:**

Our finding suggests that synergetic associations of activating and inhibitory *KIR* genes may alter the balance between these receptors and thus interfere in the progression of multibacillary leprosy.

## Author summary

Leprosy is a neglected disease with the highest worldwide prevalence, and remains a public health problem in Brazil. The innate immune mechanisms are determinants in the management of leprosy and its different clinical manifestations. Accordingly, genetic association study provides information about the contribution of host genetic factors and the environment in which the individual lives on the development of leprosy. The individuals considered most affected and associated with a major risk for developing leprosy are household contacts with an intimate relation to patients living in crowded households. For this reason, we chose the contacts as one of our control groups, since they are more exposed to infection compared to the general population. We investigated the influence of *KIR* and *HLA* genes on the susceptibility to multibacillary leprosy. Our results reinforce the importance of host genetic background in the susceptibility to leprosy demonstrating that, independent from the control group (contacts or healthy subjects) the KIR and HLA act as risk factors in the development of lepromatous and borderline leprosy.

## Introduction

Natural killer (NK) cells are crucial components of the innate immune system and provide defence against viral and microbial infections, cancer and other forms of cell stress [1]. Until recently, NK cells have been described only as killers because of their cytotoxic function in damaged cells. However, it seems that NK cells also play a major role in activating and regulating an adaptive immune response, mainly through the secretion of chemokines and inflammatory cytokines such as interferon gamma (IFN-γ), tumour necrosis factor alpha (TNF-α) and granulocyte-macrophage colony-stimulating factor (GM-CSF) [2,3]. Furthermore, a basic element in NK cell development and education is the interaction of these cells with sick and healthy cells of the human body, which occurs mostly through interactions between human leukocyte antigen (HLA) class I molecules and inhibitory or activating receptors [4,5]. NK cells have many types of cell-surface receptors to fight stressed and infected cells [6].

The receptors of NK cells containing immunoglobulin-like domains are called killer-cell immunoglobulin-like receptors (KIRs), which can be classified according to the inhibitory or activating intracellular domain. Inhibitory KIRs are codified by eight genes that prevent target cell lysis: *KIR2DL1*, *KIR2DL2*, *KIR2DL3*, *KIR2DL5A*, *KIR2DL5B*, *KIR3DL1*, *KIR3DL2* and *KIR3DL3*. On the other hand, activating KIRs promote target cell lysis and are codified by another set of genes: *KIR2DS1*, *KIR2DS2*, *KIR2DS3*, *KIR2DS4*, *KIR2DS5* and *KIR3DS1*. It should be noted that *KIR2DL4* can act as an activating or inhibitory gene. The KIR clusters also have two pseudogenes (*2DP1* and *3DP1*) and four framework genes (*3DL3*, *3DP1*, *2DL4*, *3DL2*), which are present in almost every individual [7–11]. The activity of NK cells requires interaction between a KIR and certain HLA class I ligands expressed on the surface of the cells. This interaction may protect unaltered cells from destruction or stimulate NK cell function [12–14]. The cytotoxic function of NK cells on target cells is regulated through the balance between activating and inhibitory signals that results from the interaction of KIRs and their HLA class I ligands [15,16]. Thus, it is possible that KIR molecules play a significant role in controlling the immune response during infections, which would explain the associations observed between certain KIR genes and ankylosing spondylitis [17], Chagas disease [18] and leprosy [19,20].

Leprosy is a chronic systemic granulomatous disease caused by *Mycobacterium leprae* and *M*. *lepromatosis* [21]. It is considered a major global health problem, being classified as a neglected, stigmatizing, disabling and disfiguring disease [22]. It mainly affects the peripheral nervous system, skin and other tissues such as the respiratory tract, bones and joints, eyes, nasal structures, muscles and internal organs [23–25]. According to World Health Organization (WHO) official data, Brazil had 26,875 cases of leprosy in 2017 [26]. However, it is possible that the number of hidden cases of leprosy is eight times higher than the estimated prevalence giving by WHO [27]. As result, people with leprosy are not diagnosed and the disease continues to be transmitted [27].

The development of individual clinical forms depends on the patient's immune status. The tuberculoid or paucibacillary (PB) clinical form has a predominantly cellular immune response (Th1/Th17) characterized by the production of IFN-γ, which activates CD8+ T cells, macrophages and bactericidal mechanisms [28]. These mechanisms control the growth of *M*. *leprae*, resulting in a low bacillary load or complete destruction of the bacillus. On the other hand, the lepromatous or multibacillary (MB) clinical form presents a compromised cellular immune response with a predominance of Th2/Treg, B cell activation and abundant production of IL-5, IL-6, IL-8 and IL-10 [25,28]. This characteristic immune response implies macrophagic activity suppression, excessive bacillary multiplication and spreading of infection to other organs and tissues. The lepromatous form of the disease is the most worrisome epidemiologically due to the high bacillary load in the cutaneous lesions, allowing transmission of the disease through intimate and prolonged contact [29–32]. The borderline clinical form (dimorphic) is an intermediate form that depends on the potentiality of the cellular immune response, since oscillation occurs between cellular and humoral responses [25,28,30,32].

The selection of immune response genes, which can be chosen as genetic markers for diseases, is based on the screening of proteins having a critical role in immunopathogenesis [33, 34]. Both KIRs and their HLA ligands participate in the leprosy immune response and may be important prognostic factors in a more widespread clinical form (lepromatous) of the disease. Thus, the aim of this study was to analyse the influence of genes and genotypes for KIRs and their HLA ligands on the susceptibility to, or protection from, multibacillary leprosy compared to healthy unrelated individuals (healthy subjects and household contacts).

## Methods

### Study subjects

All leprosy patients and healthy individuals were living in the north/northwest region of the State of Paraná (15th Regional Health Department of the State of Paraná, Brazil) in the period of 2015–2017. The 15th regional health department comprises 30 cities, located between latitudes 22º 41' 52" S - 23º 39' 40" S and longitudes: 51º 47' 38" W—51°59′ 25″W. This region of the State of Paraná is found in the Arenitic-Basaltic Plateau and the Southern Region of Brazil, in the transition between tropical and subtropical climates. About 25% of its territory is in the Equatorial Zone (north of the Tropic of Capricorn) and 75% in the Southern Temperate Zone. The inclusion criteria for this case–control study included 264 multibacillary leprosy patients. Multibacillary leprosy is the clinical form of the operational classification established by WHO [35]. This clinical form considers patients with more than five skin lesions and/or more than one affected nerve trunk. Furthermore, the MB form has a high bacillary load and therefore is the major source of transmission of the disease. All patients were diagnosed by experienced dermatologists at the Intermunicipal Health Consortium (CISAMUSEP) (Maringá, Paraná). The exclusion criteria include PB leprosy patients, consanguineous individuals and Asian descendent.

The controls were divided into two subgroups: a group of 280 healthy subjects, who were blood or bone marrow donors of the Regional Blood Centre of Maringá and a group of 238 healthy household contacts who had prolonged contact with the patient but were not blood relatives (spouse or family members by marriage), which characterizes them as a high risk group for contracting the disease. All controls did not have signs and symptoms of leprosy until the present time of the study.

In this study, all individuals were considered to be white Brazilians, an ethnically heterogeneous as well as mixed population due to the great miscegenation found in the population of the State of Paraná. This Brazilian region is composed mostly of individuals of white European origin (80.6%) with a small contribution of African (12.5%) and Amerindian (7%) genes [36].

### Ethics statement

The study was conducted according to the Human Research Ethics Committees from the Maringá State University, Paraná, Brazil (COPEP-UEM no. 2.424.046/2017) and from the Paraná State Department of Health (CEP-SESA/HT no. 400/2011). Both patients and controls were asked to give written informed consent before enrolling in the study.

### Genome DNA extraction

Genomic DNA samples were isolated from whole blood or buffy coat using the Biopur DNA extraction kit (Biometrix Diagnóstica, Curitiba, Paraná, Brazil), following the manufacturer`s protocol. A NanoDrop 2000 UV–Vis spectrophotometer (Thermo Fisher Scientific, Waltham, MA, USA) was used to quantify and assess DNA purity.

### *KIR* and *HLA* class I genotyping

Genotyping of *HLA-A*, *HLA-B* and *HLA-C* genes was performed by polymerase chain reaction–sequence-specific oligonucleotide probe (PCR-SSOP) using the commercial Kit LABType SSO High Definition (One Lambda Inc., Canoga Park, CA, USA) and Luminex 100 technology.

*KIR* genes were determined by PCR-SSOP using the commercial Kit LABType KIR SSO Genotyping Test (One Lambda Inc., Canoga Park, CA, USA) and Luminex 100 technology.

### Statistical analysis

The frequencies of *KIR* genes, genotypes, haplotype groups and HLA ligands were calculated by direct counting. Comparison between patients and control groups was performed using the chi-square test with Yates’ correction or Fisher’s exact test. Statistically significant *P* value (≤0.05), odds ratio (OR) and a confidence interval (CI) of 95% were analysed using OpenEpi version 3.01 software (http://www.openepi.com) [37]. The Hardy–Weinberg equilibrium (HWE) [38] was applied for *KIR2DL2/3*, *KIR3DL1/S1* and HLA using Arlequin software version 3.1.30. Individuals were classified into four groups [7] according to their HLA ligands: HLA-A3 or A11, HLA-Bw4 [10], HLA-C group 1 (C1) and HLA-C group 2 (C2) [39,40].

Adjusted odds ratios (ORs) were estimated using multiple logistic regressions after inclusion of matching variables (*KIR* genes and HLA class I ligands). All statistical analyses were performed using the Stata software version 12.0 (Stata Corporation, College Station, TX, USA and *P* adjusted < 0.05 was considered statistically significant.

The genotype ID and *KIR* haplotype groups (AA, Bx) were obtained from the Allele Frequency Net Database (http://www.allelefrequencies.net/kir6001a.asp)[41]. *KIR* genotypes were classified as AA and Bx haplotype groups. The haplotype B group is characterized by the presence of any of these genes: *2DL2*, *2DL5*, *3DS1*, *2DS1*, *2DS2*, *2DS3*, and *2DS5*. However, KIR genotypes that do not have these genes are considered as AA haplotype, and present predominantly inhibitory and framework genes. The B haplotype group was considered only when it was not possible to distinguish between AB and BB genotypes.

## Results

### Case-control (participants) characteristics

This case–control study enrolled 264 multibacillary leprosy unrelated patients: 158 (59.8%) men and 106 (40.2%) women, with a mean (SD) age of 54.3 (±14.6) years. Multibacillary patients were also classified according to the Madrid (1953) classification [42] into two distinct groups: 143 (54.2%) were diagnosed with lepromatous leprosy (LL) or Virchowian leprosy (VL) and 121 (45.8%) with borderline leprosy (BL) or dimorphic leprosy (DL). The demographic and clinical characteristics of the studied subjects are shown in Table 1.

**Table 1:** Characteristics of the studied population of leprosy patients and controls (healthy household contacts and healthy subjects) according to clinical forms, age, and gender.

		MB leprosy N = 264	Lepromatous N = 143	Borderline N = 121	Contacts N = 238	Healthy subjects N = 280
**Age**	Mean	54.3	53.4	55.6	60.3	35.8
	SD	±14.6	±14.3	±14.9	±17.4	±10.6
		**n (%)**	**n (%)**	**n (%)**	**n (%)**	**n (%)**
**Gender**	Male	158 (59.8)	106 (74.1)	52 (42.9)	122 (51.3)	126 (45.0)
	Female	106 (40.2)	37 (25.9)	69 (57.1)	116 (48.7)	154 (55.0)

MB: multibacillary leprosy (lepromatous leprosy + borderline leprosy); N: number of individuals; SD: Standard Deviation; Contacts: healthy household contacts.

Regarding to the controls, a group of 280 healthy subjects comprised: 126 (45%) men and 154 (55%) women, with a mean (SD) age of 35.8 (±10.6) years. In addition, a second control group of 238 healthy household contacts was formed by: 122 (51.3%) men and 116 (48.7%) women, with a mean (SD) age of 60.3 (±17.4) years. Control’s age and gender distribution are summarized in Table 1.

### Frequency of *KIR* and *HLA* ligand genes in patients and control groups

The distribution of *HLA* and *KIR* genotype frequencies in the population studied was according to HWE. The *KIR* gene frequencies for the MB leprosy patients, their clinical subgroups and the control groups are shown in S1 Table in Supporting Information. None of the *KIR* genes were associated with leprosy when compared to the control groups. Comparisons of HLA class I ligand (HLA-A*03/A*11, HLA-Bw4, HLA-C1 and HLA-C2) frequencies are given in Table 2. The Borderline Leprosy (BL) group revealed a significant increase of the HLA-A variants (A3 or A11) compared to the healthy subjects (HS) (36.4% vs. 25.0%, *P* = 0.028, OR = 1.71, 95% CI = 1.08–2.71), even after multiple logistic regression (Adjusted OR = 2.24, *P* = 0.035).

**Table 2:** Frequencies of *HLA* class I (*KIR* ligands) in multibacillary leprosy patients, clinical subgroups and controls (healthy household contacts and healthy subjects).

HLA Ligands	MB leprosy N = 264 n (%)	Lepromatous N = 143 n (%)	Borderline N = 121 n (%)	Contacts N = 238 n (%)	Healthy subjects N = 280 n (%)	Unadjusted OR (95% IC)	*P*	Adjusted OR (95% IC)	*P*
HLA-A*03 or HLA-A*11	73 (27.7)	29 (20.3)	44 (36.4)	59 (24.8)	70 (25.0)	1.73 (1.08–2.78)	0.030^a^	1.35 (0.67–2.76)	0.401^a^
						1.71 (1.08–2.71)	0.028^b^	2.24 (1.06–4.77)	0.035^b^
						0.44 (0.25–0.77)	0.005^c^	0.43 (0.17–1.05)	0.065^c^
HLA-A*03	50 (18.9)	20 (14.0)	30 (24.8)	35 (14.7)	53 (18.9)	1.91 (1.10–3.30)	0.027^a^	1.04 (0.38–2.84)	0.944^a^
						0.49 (0.26–0.92)	0.037^c^	0.68 (0.29–1.54)	0.358 ^c^
HLA-A*11	27 (10.2)	10 (7.0)	17 (14.0)	24 (10.1)	21 (7.5)				
**Bw4**	201 (76.1)	108 (75.5)	93 (76.9)	194 (81.5)	219 (78.2)				
**Bw4-Bw4**	77 (29.2)	40 (28.0)	37 (30.6)	64 (26.9)	89 (31.8)				
**C1**	207 (78.4)	116 (81.1)	91 (75.2)	187 (78.6)	223 (79.6)				
**C1/C1**	85 (32.2)	46 (32.2)	39 (32.2)	69 (29.0)	89 (31.8)				
**C2**	175 (66.3)	95 (66.4)	80 (66.1)	169 (71.0)	191 (68.2)				
**C2/C2**	53 (20.1)	25 (17.5)	28 (23.1)	51 (21.4)	57 (20.4)				
**C1/C2**	122 (46.2)	70 (49.0)	52 (43.0)	118 (49.6)	134 (47.9)				

MB: multibacillary leprosy (lepromatous leprosy + borderline leprosy); N: number of individuals; n: number of individuals with the HLA ligands; OR: odds ratio; 95% CI: confidence interval; Adjusted OR: multiple logistic regression.

^**a**^Borderline leprosy patients *vs*. contacts

^**b**^Borderline leprosy patients *vs*. healthy subjects

^**c**^Lepromatous leprosy patients *vs*. borderline leprosy patients (All *P* adjusted by A3/A11 and A3 ligands).

**Bw4:** A9, A*23, A*24, A*25, A*32, B5, B*13, B*17, B*27, B*37, B*38, B*44, B*47, B*49, B*51, B*52, B*53, B*57, B*58, B*59, B*63 and B*77;

**Group C1:** C*01, C*03, C*07, C*08, C*12, C*13, C*14, C*16:*01*, C*16:03 and C*16:*04*

**Group C2:** C*02, C*04, C*05, C*06, C*15, C*16:*02*, C*17 and C*18.

In addition, the A*03 or A*011 variants were also significantly increased in borderline form in comparison to household contacts (HC) (36.4% vs. 24.8%, *P* = 0.030, OR = 1.73, 95% CI = 1.08–2.78), and in comparison to lepromatous leprosy (LL) (*P* = 0.005, OR = 0.44, 95% CI = 0.25–0.77), although for both of them the significance was lost after *P*-value adjustment (*P* = 0.401 and 0.065, respectively).

Furthermore, when HLA ligand A3 and A11 are analysed separately, it was observed an association for the variant A3 between clinical form BL and contacts (24.8% vs. 14.7%, *P* = 0.027, OR = 1.91, 95% CI = 1.10–3.30), and the clinical forms LL and BL (14.0% vs. 24.8%, *P* = 0.037, OR = 0.49, 95% CI = 0.26–0.92). However, for all of them the significance was lost after multiple logistic regression. Other ligands showed no significant differences for any clinical forms compared to the control groups.

### Frequency of KIR-HLA combinations in patients and control groups

To evaluate the *KIR* and their HLA ligand genes in the outcome of MB leprosy patients, the frequencies of the *KIR-HLA* combinations were compared among patients, clinical forms and controls. The significant results are shown in Table 3 (complete analysis results can be found in S2 Table). All results have kept significance after multiple logistic regression, except the association *3DS1/Bw4*- for BL clinical form compared to the contacts (*P* = 0.048, Adjusted *P* = 0.058).

**Table 3:** Significant associations of *KIR* genes and *HLA* ligands in multibacillary leprosy patients, their clinical subgroups and controls (healthy household contacts and healthy subjects).

*KIR*–HLA ligands	MB leprosy N = 264 n (%)	Lepromatous N = 143 n (%)	Borderline N = 121 n (%)	Contacts N = 238 n (%)	Healthy subjects N = 280 n (%)	Unadjusted OR (95% IC)	*P*	Adjusted OR (95% IC)	*P*
***3DS1*/Bw4-**	28 (10.6)	12 (8.4)	16 (13.2)	16(6.7)	22 (7.9)	2.11 (1.01–4.39)	0.048^a^	2.06 (0.98–4.36)	0.058^a^
***2DS5/C2+***	78 (29.6)	46 (32.2)	32 (26.4)	63 (26.5)	61 (21.8)	1.50 (1.02–2.21)	0.048^b^	1.50 (1.02–2.21)	0.048 ^b^
						1.70 (1.08–2.67)	0.027^c^	1.62 (1.03–2.56)	0.037^c^
***3DL2/*A3/A11+**	73 (27.7)	29 (20.3)	44 (36.4)	59 (24.8)	70 (25.0)	1.73 (1.08–2.78)	0.030^a^	1.84 (1.13–2.97)	0.014^a^
						1.71 (1.08–2.71)	0.028^d^	1.71 (1.08–2.71)	0.028^d^
						0.44 (0.25–0.77)	0.005^e^	0.42 (0.24–0.73)	<0.01^e^
***2DL2/2DL2*/C1C2+**	17 (6.4)	12 (8.4)	5 (4.1)	11 (4.6)	8 (2.9)	3.11 (1.24–7.80)	0.021^e^	2.85 (1.13–7.22)	0.027^c^
***2DL3/2DL3*/C1C1+**	45 (17.0)	19 (13.3)	26 (21.5)	28 (11.8)	45 (16.1)	2.05(1.14–3.69)	0.022^a^	2.13 (1.17–3.87)	0.014^a^
***2DS1*/C2-**	35(13.2)	13(9.1)	22(18.2)	32 (13.4)	38 (13.6)	0.45(0.21–0.93)	0.032^e^	0.45 (0.21–0.93)	0.046 ^e^
***2DS1/2DL1/C2-***	35(13.2)	13(9.1)	22 (18.2)	30 (12.6)	37 (13.2)	0.45(0.21–0.93)	0.032^e^	0.41 (0.19–0.86)	0.018^e^

MB = multibacillary leprosy (lepromatous leprosy + borderline leprosy); N: number of individuals; n: number of individuals with the *KIR* genes their *HLA* ligands; OR: odds ratio; 95% CI: confidence interval; Adjusted OR: multiple logistic regression.

^**a**^Borderline leprosy patients *vs*. contacts (Adjusted by *3DS1*/Bw4-, *3DL2/*A3/A11+,*2DL3/2DL3*/C1C1+)

^**b**^MB leprosy patients *vs*. healthy subjects (Adjusted by *2DS5/C2+*)

^**c**^Lepromatous leprosy patients *vs*. healthy subjects (Adjusted by *2DS5/C2+*,*2DL2/2DL2*/C1C2+)

^**d**^Borderline leprosy patients *vs*. healthy subjects (Adjusted by *3DL2/*A3/A11+)

^**e**^Lepromatous leprosy patients *vs*. borderline leprosy patients (Adjusted by *3DL2/*A3/A11+, *2DS1*/C2-,*2DS1/2DL1/C2-*).

The higher frequency of the combination of *KIR2DS5* activating gene with its C2 ligand (*KIR2DS5/C2+*) was associated with MB leprosy patients when compared to healthy subjects (29.6% vs. 21.8%, *P* = 0.048, adjusted OR = 1.50, 95% CI = 1.02–2.21). *KIR2DS5/C2+* also showed an increased risk for LL patients compared to healthy subjects (32.2% vs. 21.8%, *P* = 0.037, adjusted OR = 1.62, 95% CI = 1.03–2.56).

In contrast, analysis of *KIR3DL2* in the presence of A3/A11 ligands showed a risk association by its increased frequency in BL patients compared to contacts (36.4% vs. 24.8%, *P* = 0.014, adjusted OR = 1.84, 95% CI = 1.13–2.97) and also over HS (36.4% vs. 25.0%, *P* = 0.028, adjusted OR = 1.71, 95% CI = 1.08–2.71). However, *KIR3DL2/A3/A11****+*** showed an increased protection in LL patients when compared to BL patients (20.3% vs. 36.4%, *P* <0.01, adjusted OR = 0.42, 95% CI = 0.24–0.73).

The frequencies of *KIR* genes were also analysed for homozygosis/heterozygosis and the presence/absence of their HLA ligands. The significant results are shown in Table 3. Among the inhibitory genes, *KIR2DL2/2DL2/C1/C2+* had higher frequency in the LL clinical form to the HS (8.4% vs. 2.9%, *P* = 0.027; adjusted OR = 2.85; 95% CI = 1.13–7.22). In the analysis of homozygous *KIR2DL3* with its homozygous C1 ligand (*KIR2DL3/2DL3/C1/C1+*), the frequency was higher in the BL patients than in contacts (21.5% vs. 11.8%, *P* = 0.014, adjusted OR = 2.13, 95% CI = 1.17–3.87).

The lower frequency of the combination *KIR2DS1+/C2−* for LL compared to BL was statistically significant (9.1% vs. 18.2%; *P* = 0.046, adjusted OR = 0.45, 95% CI = 0.21–0.93). Furthermore, this association was maintained in the presence of its inhibitory homologue *KIR2DL1* and in the absence of the C2 ligand (*KIR2DS1/KIR2DL1/C2***−**) (9.1% vs. 18.2%; *P* = 0.018, adjusted OR = 0.41, 95% CI = 0.19–0.86).

### Frequency of *KIR* genotypes in patients and control groups

The results of *KIR* genotypes can be seen in S3 Table in Supporting Information. A total of 68 distinct *KIR* genotypes were identified in 782 individuals. We found 23 common genotypes between the MB leprosy patients and the controls. Furthermore, we identified 12 genotypes present only in MB leprosy patients; one of these genotypes has no assigned ID yet (NAS0). In addition, the contacts presented exclusively 12 genotypes, one of which has not been assigned an ID yet (NAS1). Already, the healthy subjects presented exclusively 9 genotypes.

Genotype ID 1 was significantly decreased in the MB leprosy patients compared to the HS (25.0% vs. 33.2%, *P* = 0.044, OR = 0.67, 95% CI = 0.46–0.97). Moreover, genotype 1 was shown to act as a protection factor for the healthy subjects compared to the clinical form BL (33.2% vs. 22.3%, *P* = 0.03, OR = 0.57, 95% CI = 0.35–0.94). In addition, genotype ID 3 showed a protection against BL compared to contacts (0.8% vs. 5.9%, *P* = 0.047, OR = 0.13, 95% CI = 0.003–0.89). However, genotype ID 3 was significantly increased in the LL patients compared to BL patients (6.3% vs. 0.8%, *P* = 0.046, OR = 8.06, 95% CI = 1.08–undefined).

## Discussion

This study provides information about the contribution of host genetic factors and the environment in which the individual lives on the development of leprosy. Our analyses reveal new and important associations but also confirm data already published. To the best of our knowledge, this is the first study to use two different control groups: a group of household contacts with leprosy patients that had intimate and prolonged contact; and another group of unrelated individuals, recruited as blood or bone marrow donors, traditionally used in case–control studies. The individuals considered most affected and associated with a major risk for developing leprosy are household contacts with an intimate relation to patients living in crowded households. For this reason, we chose the contacts as one of our control groups, since they are more exposed to infection compared to the general population. Besides intimate contact between the HHC and MB patients, there is also evidence that increased age, poor health and socioeconomic conditions are associated with an increased risk of the disease [31,43].

But what lies behind the immunopathogenesis of leprosy? New evidence suggests that innate immune mechanisms are determinants in the management of leprosy and its different clinical manifestations. Among the elements of this immune response involved in the pathogenesis of the disease are toll-like receptors, vitamin D receptors, NK cells, macrophages, dendritic cells and neutrophils [34]. Natural killer cells are vital cells that recognize and eliminate neoplastic and infected cells by secreting perforins and granzymes. The balance between activating and inhibitory signals regulates the cytotoxic function of NK cells [4,44,45]. Recent studies have shown that the imbalance between activating and inhibitory *KIR* genes is associated with late activation of NK cells, favouring the intracellular proliferation of *M*. *leprae*.

Corroborating data already described in the literature, our results are strengthened by the analysis of Franceschi et al. (2008) [20]. These authors described ligands A3 and/or A11 as susceptibility factors in the development of BL. Our results confirmed that these variants are in fact a risk to this clinical form even when compared to the healthy subjects. We had similar results when analysing the combination of *KIR3DL2* and HLA-A*03/A*11 genes. This *KIR* framework gene in the presence of its ligand was revealed to be a susceptibility factor for BL compared to contacts (adjust OR = 1.84) and HS (adjust OR = 1.71) (Table 3). However, this susceptibility must be due to the presence of A*03/A*11 ligands only (Table 2), and not by the presence of the *KIR3DL2* gene, which is present in approximately 100% of the different world populations [46]. In addition, KIR3DL2 is considered to have a low inhibitory ability and the interaction with its ligand HLA-A3 and HLA-A11 is extremely peptide-specific present in the groove [47,48], which allows the epitopes A3 and A11 to present peptides from different infectious diseases [48].

Our results with *KIR2DS5/C2+* suggest a controversial association with the disease and its more widespread clinical form. This fact can be explained by the variability found in the affinity of *KIR2DS5* for its ligand, caused by the highly polymorphic sequence of this gene. This variable avidity for the HLA ligand can modulate activation of NK cells, which affects progression of the disease or healing [49]. It is therefore important to perform an analysis of *KIR2DS5* and HLA-C2 allotypes in our LL patients to define whether our results demonstrate a new tendency in this infectious disease or if it is due to the polymorphic characteristics of our study subjects. Furthermore, recent studies have reported a risk association of *KIR2SD5* with colorectal cancer patients in Caucasian Brazilian [50] and Korean populations [51] and Lebanese patients with multiple myeloma [52]. In contrast, Blokhuis et al. (2017) [49] reported that the allele KIR2DS5*006 protects pregnant women in Uganda from pre-eclampsia. Thus, we can observe that *KIR2DS5* may play a different role in several different diseases.

Although Jarduli et al. (2014) [19] have not found an association of the homozygous *KIR2DL2* gene in the presence of the heterozygous HLA-C group C1/C2 (*KIR2DL2*/*KIR2DL2/C1/C2*) with leprosy, our analysis has shown a risk to the clinical form LL compared to the healthy subjects. We also showed that *KIR2DL3/KIR2DL3/C1/C1* is strongly increased in BL patients over the contacts, but previous studies in Southern Brazil [20] and the Mid-West [19] did not show a significant association between *KIR2DL3/KIR2DL3/C1/C1* and the susceptibility of BL. In addition, it has already been shown that KIR2DL2 receptor binds stronger than KIR2DL3 to HLA-C ligands, which could explain why we have the association between KIR2DL2 and LL clinical form, while KIR2DL3 associates with borderline leprosy, a less severe form of the disease [4,6].

When we analyse the frequency between patients with the polar form of the disease (LL) compared with the unstable (BL) ones, a high frequency *2DS1/C2–* and *2DS1/2DL1/C2–* was observed in BL patients. These KIR-HLA combinations appear to play an important role in resistance to leprosy, avoiding progression to the most severe form of the disease. Probably, in the absence of an inhibitory stimulus the NK cells tend to become activated, because of the triggering of other activation receptors, as the 2DS5 and 3DS1 that are in linkage disequilibrium with 2DS1 [42]. This could change the NK function to become activated, which favours an immune response, so the disease evolves to the BL form rather than the LL form [53].

Regarding KIR genotype profiles, our study demonstrated for the first time in the literature that genotype ID1 seem to play a protective role in MB patients, mainly to its BL clinical form. Once genotype ID 1 is composed of six genes that encode the inhibitory receptors and only one activating gene (*KIR2DS4*), it could seem contradictory that with only one activating receptor, the individual has a better answer against *M*. *leprae*, when comparing HS versus BL clinical form (33.2% vs. 22.3%, *P* = 0.03, OR = 0.57, 95% CI = 0.35–0.94). However, we must to keep in mind that KIR receptors are not the only activating receptor in NK cells, as follows: FcγRIIIA (CD16), natural cytotoxicity receptors (NKp30, NKp44, NKp46), NKG2D (CD314) and NKG2C (CD159C) [1]). With respect to genotype ID 3, our analysis showed a protection for contacts compared to BL clinical form, which could be explained by the presence of almost all KIR activating receptors (2DS4, 3DS1, 2DS1, 2DS2, 2DS5), only 2DS3 receptor is not present. Despite some studies suggesting susceptibility or protection, it is still difficult to fully comprehend the role of KIR and their HLA ligands in leprosy. One of the reasons is the reduced number of studies that have analysed large cohorts with clinical forms of leprosy and control groups. Furthermore, *KIR* genes are not the only receptors acting in NK cells, as well as, they are not exclusive receptors of these cells. All of the known KIRs can be expressed by natural killer T cells (NKT), subsets of γδ+ T cells, and αβ+ T cells; however, in most individuals, they are expressed by CD8+ cells, but are infrequent in CD4+ cells [54]. Thus, KIR receptors could play a role in physiopathology of leprosy through the action of these cells, other than NK cells, but the exact role of all those cells have not been defined till date [30,55].

**Fig 1:**
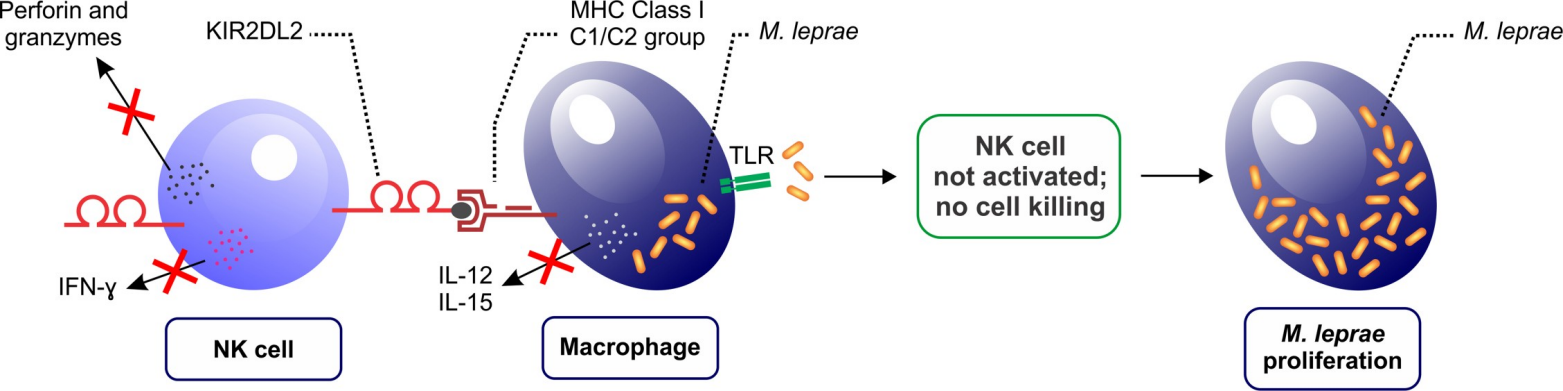
Inhibitory KIR receptors and their ligands HLA-C1/C2 may play a critical role in the development of leprosy and its clinical forms. The prevalence of inhibitory signals (e.g. KIR2DL2/C1/C2) on NK cells could lead to attenuation of cell activity, which impairs the cytotoxic or immunoregulatory function of NK cells by inhibiting the secretion of perforins, granzymes and IFN-γ. *Mycobacterium leprae* also inhibits the synthesis of interleukins (IL-12 and IL-15) by macrophages that are infected. The lack of IFN-γ and interleukins synthesized by NK cells and macrophages, respectively, will increase the bacillary load of *M*. *leprae*, leading to the development of MB clinical forms. TLR: toll-like receptor; IFN-γ: interferon gamma.

In conclusion, we highlight the new associations found in this study, where *KIR2DL2/2DL2/C1/C2+* and *KIR2DL3/2DL3/C1/C1+* are indicated to be important risk factors for developing lepromatous and borderline leprosy, respectively. These results of association suggest that in the presence of inhibitory KIR and their ligands HLA-C1/C2, NK cells could not exert a cytotoxic or immunoregulatory role, and the lack of IFN-γ synthesis would enable an increase in the bacillary load, leading to the development of MB clinical forms (Fig 1). Concerning to *3DL2/A3/A11+*, our study demonstrated that independent of control group (contacts or healthy subjects), this KIR receptor and its ligand act as a risk factor for the borderline clinical form. Our findings suggest that synergetic associations of activating and inhibitory *KIR* genes may alter the balance between these receptors, and thus interfere in progression of the disease, either through the control of cytokines release or cytotoxic profile of NK and CD8+ T cells.

## Supporting information

S1 Table Distribution of *KIR* gene frequencies in multibacillary leprosy patients, clinical subgroups and controls (healthy household contacts and healthy subjects). (DOCX)

S2 Table Distribution of *KIR* genes and their respective HLA ligands in controls (healthy household contacts and healthy subjects), MB leprosy patients and their clinical subgroups. (DOCX)

S3 Table *KIR* genotype profiles of multibacillary leprosy patients, their clinical subgroups and controls (healthy household contacts and healthy subjects). (DOCX)

S4 Table STROBE Statement—Checklist of items that should be included in reports of case-control studies. (DOCX)
