# Supplementary material for: The impact of KIR/HLA genes on the risk of developing multibacillary leprosy
Source: PLoS Negl Trop Dis. 2019 Sep 16;13(9):e0007696. doi: 10.1371/journal.pntd.0007696 (PMC6762192; doi:10.1371/journal.pntd.0007696)
Supplement: S1 Table — (DOCX) [file pntd.0007696.s001.docx]

**Supporting Information**

**Table S1**. Distribution of *KIR* gene frequencies in multibacillary leprosy patients, clinical subgroups and controls (healthy household contacts and healthy subjects)

| **Genes#** | **MB leprosy**  **N = 264**  **n (%)** | **Lepromatous**  **N = 143**  **n (%)** | **Borderline**  **N = 121**  **n (%)** | **Contacts**  **N = 238**  **n (%)** | **Healthy subjects**  **N = 280**  **n (%)** |
| --- | --- | --- | --- | --- | --- |
| **Inhibitory *KIR*** |  |  |  |  |  |
| ***KIR2DL1*** | 254 (96.2) | 136 (95.1) | 118 (97.5) | 228 (95.8) | 272 (97.1) |
| ***KIR2DL2*** | 133 (50.4) | 75 (52.4) | 58 (47.9) | 134 (56.3) | 133 (47.5) |
| ***KIR2DL3*** | 238 (90.2) | 126 (88.1) | 112 (92.6) | 218 (91.6) | 249 (88.9) |
| ***KIR2DL4*⁺*** | 264 (100) | 143 (100) | 121 (100) | 236 (99.2) | 280 (100) |
| ***KIR2DL5*** | 149 (56.4) | 78 (54.5) | 71 (58.7) | 134 (56.3) | 147 (52.5) |
| ***KIR3DL1*** | 244 (92.4) | 134 (93.7) | 110 (90.9) | 222 (93.3) | 264 (94.3) |
| ***KIR3DL2⁺*** | 264 (100) | 143 (100) | 121 (100) | 238 (100) | 280 (100) |
| ***KIR3DL3⁺*** | 264 (100) | 143 (100) | 121 (100) | 238 (100) | 280 (100) |
| **Activating *KIR*** |  |  |  |  |  |
| ***KIR2DS1*** | 115 (43.6) | 57 (39.9) | 58 (47.9) | 99 (41.6) | 113 (40.4) |
| ***KIR2DS2*** | 132 (50.0) | 71 (49.7) | 61 (50.4) | 131 (55.0) | 133 (47.5) |
| ***KIR2DS3*** | 75 (28.4) | 38 (26.6) | 37 (30.6) | 75 (31.5) | 76 (27.1) |
| ***KIR2DS4*** | 239 (90.5) | 131 (91.6) | 109 (90.1) | 220 (92.4) | 263 (93.9) |
| ***KIR2DS5*** | 107 (40.5) | 57 (39.9) | 50 (41.3) | 90 (37.8) | 96 (34.3) |
| ***KIR3DS1*** | 108 (40.9) | 56 (39.2) | 52 (43.0) | 92 (38.7) | 109 (38.9) |
| **Pseudogene *KIR*** |  |  |  |  |  |
| ***KIR2DP1*** | 257 (97.3) | 137 (95.8) | 120 (99.2) | 231 (97.1) | 271 (96.8) |
| ***KIR3DP1⁺*** | 264 (100) | 143 (100) | 121 (100) | 236 (99.2) | 280 (100) |

**KIR2DL4*: activator or inhibitor; ***⁺****KIR2DL4, KIR3DL2, KIR3DL3* and *KIR3DP1*: framework genes;

MB = multibacillary leprosy (lepromatous leprosy + borderline leprosy); N: number of individuals; n: number of individuals with the gene.

**^#^**No significant difference was observed in the distribution of *KIR* genes between control groups and leprosy MB.
