# Supplementary material for: The impact of KIR/HLA genes on the risk of developing multibacillary leprosy
Source: PLoS Negl Trop Dis. 2019 Sep 16;13(9):e0007696. doi: 10.1371/journal.pntd.0007696 (PMC6762192; doi:10.1371/journal.pntd.0007696)
Supplement: S2 Table — (DOCX) [file pntd.0007696.s002.docx]

**Table S2.** Distribution of *KIR* genes and their respective HLA ligands in controls (healthy household contacts and healthy subjects), MB leprosy patients and their clinical subgroups.

| *KIR* – HLA ligands | MB leprosy  N = 264  n (%) | Lepromatous  N = 143  n (%) | Borderline  N = 121  n (%) | Contacts  N = 238  n (%) | Healthy subjects  N = 280  n (%) |
| --- | --- | --- | --- | --- | --- |
| *2DL1/*C2+ | 166 (62.9) | 89 (62.2) | 77 (63.6) | 162 (68.1) | 186 (66.4) |
| *2DL1/C2-* | 88 (33.3) | 47 (32.8) | 41 (33.8) | 66 (27.7) | 86 (30.7) |
| *2DL1/*C2C2+ | 52 (19.7) | 25 (17.5) | 27 (22.3) | 49 (20.6) | 55 (19.6) |
| *2DL2*/C1+ | 105 (39.8) | 64 (44.8) | 41 (33.9) | 102 (42.9) | 104 (37.1) |
| *2DL2*/C1- | 29 (11.0) | 10 (7.0) | 19 (15.7) | 32 (13.4) | 29 (10.4) |
| *2DL2*/C1C1+ | 40 (15.2) | 27 (18.9) | 13 (10.7) | 41 (17.2) | 44 (15.7) |
| *2DL3/*C1+ | 185 (70.1) | 100 (69.9) | 85 (70.2) | 171 (71.8) | 202 (72.1) |
| *2DL3/*C1- | 53 (20.1) | 26 (18.2) | 27(22.3) | 47 (19.7) | 47 (16.8) |
| *2DL3*/C1C1+ | 80 (30.3) | 42 (29.4) | 38 (31.4) | 64(26.9) | 76 (27.1) |
| *3DL1/*Bw4+ | 189 (71.6) | 102 (71.3) | 87 (71.9) | 182 (76.5) | 206 (73.6) |
| *3DL1/*Bw4- | 55 (20.8) | 32 (22.4) | 23 (19.0) | 40 (16.8) | 58 (20.7) |
| *3DL1/*Bw4Bw4+ | 72 (27.3) | 36 (25.2) | 36 (29.8) | 60 (25.2) | 81 (28.9) |
| *3DL2/*A3/A11+ | 73 (27.7) | 29 (20.3) **^c^** | 44 (36.4)**^a b c^** | 59 (24.8) **^a^** | 70 (25.0) **^b^** |
| *2DS1*/C2+ | 80 (30.3) | 44 (30.8) | 36 (29.8) | 67 (28.2) | 75 (26.8) |
| *2DS1*/C2- | 33 (12.5) | 13 (9.1) **^d^** | 22 (18.2) **^d^** | 32 (13.4) | 38 (13.6) |
| *2DS1*/C2C2+ | 21 (8.0) | 13 (9.1) | 8 (6.6) | 26 (10.9) | 27 (9.6) |
| *2DS2*/C1+ | 104 (39.4) | 60 (42.0) | 44 (36.4) | 99 (41.6) | 104 (37.1) |
| *2DS2*/C1- | 28 (10.6) | 11 (7.7) | 17 (14.0) | 32 (13.4) | 29 (10.4) |
| *2DS2/*C1C1+ | 42 (15.9) | 26 (18.2) | 16 (13.2) | 40 (16.8) | 42 (15.0) |
| *2DS3/C1+* | 57 (21.6) | 31 (21.7) | 26 (21.5) | 60 (25.2) | 56 (20.0) |
| *2DS3/C1-* | 18 (6.1) | 7 (4.2) | 11 (8.3) | 15 (6.3) | 20 (7.1) |
| *2DS3/C1C1+* | 23 (8.7) | 14 (9.8) | 9 (7.4) | 20 (8.4) | 23 (8.2) |
| *3DS1*/Bw4+ | 80 (30.3) | 44 (30.8) | 36 (29.8) | 76 (31.9) | 87 (31.1) |
| *3DS1*/Bw4- | 28 (10.6) | 12 (8.4) | 16 (13.2) **^e^** | 16 (6.7) **^e^** | 22 (7.9) |
| *3DS1*/Bw4Bw4+ | 31 (11.7) | 18 (12.6) | 13 (10.7) | 19 (8.0) | 40 (14.3) |
| *2DS5/C2+* | 78 (29.5) **^f^** | 46 (32.2) **^g^** | 32 (26.4) | 63 (26.5) | 61 (21.8) **^f g^** |
| *2DS5/C2-* | 29 (11.0) | 11 (7.7) | 18 (14.9) | 27 (11.3) | 35 (12.5) |
| *2DS5/C2C2+* | 16 (6.1) | 10 (7.0) | 6 (5.0) | 24 (10.1) | 21 (7.5) |
| *2DL2/2DL2*/C1+ | 22 (8.3) | 16 (11.2) | 6 (5.0) | 16 (6.7) | 21 (7.5) |
| *2DL2/2DL2*/C1- | 4 (1.5) | 1 (0.7) | 3 (2.5) | 4 (1.7) | 9 (3.2) |
| *2DL2/2DL2*/C1C1+ | 5 (1.9) | 4 (2.8) | 1 (0.8) | 5 (2.1) | 13 (4.6) |
| *2DL2/2DL2*/C1C2+ | 17 (6.4) | 12 (8.4) **^h^** | 5 (4.1) | 11 (4.6) | 8 (2.9) **^h^** |
| *2DL3/2DL3*/C1+ | 102 (38.6) | 52 (36.4) | 50 (41.3) | 85(35.7) | 119 (42.5) |
| *2DL3/2DL3*/C1- | 29 (11.0) | 16 (11.2) | 13 (10.7) | 19 (8.0) | 27 (9.6) |
| *2DL3/2DL3*/C1C1+ | 45 (17.0) | 19 (13.3) | 26 (21.5) **^i^** | 28 (11.8) **^i^** | 45 (16.1) |
| *2DL3/2DL3*/C1C2+ | 57 (21.6) | 33 (23.1) | 24 (19.8) | 57 (23.9) | 74 (26.4) |
| *2DL2/2DL3*/C1+ | 83 (31.4) | 48 (33.6) | 35 (28.9) | 86 (36.1) | 83 (29.6) |
| *2DL2/2DL3*/C1- | 24 (9.1) | 10 (7.0) | 14 (11.6) | 28(11.8) | 20 (7.14) |
| *2DL2/2DL3*/C1C1+ | 35 (13.3) | 23 (16.1) | 12 (9.9) | 36 (15.1) | 31 (11.1) |
| *2DL2/2DL3*/C1C2+ | 48 (18.2) | 25 (17.5) | 23 (19.0) | 50 (21.0) | 52 (18.6) |
| *2DS2/2DL2*/C1+ | 101 (38.3) | 60 (42.0) | 41 (33.9) | 98 (41.2) | 101 (36.1) |
| *2DS2/2DL2*/C1- | 28 (10.6) | 11 (7.7) | 17 (14.0) | 31 (13.0) | 28 (10.0) |
| *2DS2/2DL2*/C1C1+ | 39 (14.8) | 26 (18.2) | 13 (10.7) | 40 (16.8) | 42 (15.0) |
| *2DS2/2DL2*/C1C2+ | 62 (23.5) | 34 (23.8) | 28 (23.1) | 58(24.4) | 59 (21.1) |
| *2DS2+ /2DL2-*/C1+ | 3 (1.1) | 0 | 3 (2.5) | 1 (0.4) | 3 (1.1) |
| *2DS2- /2DL2+* /C1+ | 4 (1.5) | 4 (2.8) | 0 | 4 (1.7) | 3 (1.1) |
| *2DS1/2DL1/C2+* | 76 (28.8) | 42 (29.4) | 34 (28.1) | 64 (26.9) | 73 (26.1) |
| *2DS1/2DL1/C2-* | 33 (12.5) | 12 (8.4) **^j^** | 21 (17.4) **^j^** | 30 (12.6) | 37 (13.2) |
| *2DS1/2DL1/C2C2+* | 21 (8.0) | 13 (9.1) | 8 (6.6) | 25 (10.5) | 25 (8.9) |
| *2DS1/2DL1/C1C2+* | 55 (20.8) | 29 (20.3) | 26 (21.5) | 39 (16.4) | 48 (17.1) |
| *2DS1+ /2DL1- / C2+* | 4 (1.5) | 2 (1.4) | 2 (1.7) | 3 (1.3) | 2 (0.7) |
| *2DS1- /2DL1+ / C2+* | 90 (34.1) | 47 (32.9) | 43 (35.5) | 98 (41.2) | 113 (40.4) |
| *2DS2/2DL3/C1+* | 82 (31.1) | 44 (30.8) | 38 (31.4) | 83 (34.9) | 83 (29.6) |
| *2DS2/2DL3/C1-* | 24 (9.1) | 10 (7.0) | 14 (11.6) | 28 (11.8) | 19 (6.8) |
| *2DS2/2DL3/C1C1+* | 37 (14.0) | 22 (15.4) | 15 (12.4) | 35 (14.7) | 29 (10.4) |
| *2DS2/2DL3/C1C2+* | 45 (17.0) | 22 (15.4) | 23 (19.0) | 48 (20.2) | 54 (19.3) |
| *2DS2+ / 2DL3- / C1+* | 22 (8.3) | 16 (11.2) | 6 (5.0) | 16 (6.7) | 21 (7.5) |
| *2DS2- / 2DL3+ / C1+* | 103 (39.0) | 56 (39.2) | 47 (38.8) | 88 (37.0) | 119 (42.5) |
| *2DS3/2DL3/C1+* | 44 (16.7) | 22 (15.4) | 22 (18.2) | 49 (20.6) | 41 (14.6) |
| *2DS3/2DL3/C1-* | 14 (5.3) | 6 (4.2) | 8 (6.6) | 13(5.5) | 12 (4.3) |
| *2DS3/2DL3/C1/C1+* | 19 (7.2) | 11 (7.7) | 8 (6.6) | 17 (7.1) | 13 (4.6) |
| *2DS3+/2DL3- /C1+* | 13 (4.9) | 9 (6.3) | 4 (3.3) | 11 (4.6) | 15 (5.4) |
| *2DS3- /2DL3+ /C1+* | 141 (53.4) | 78 (54.5) | 63 (52.1) | 122 (51.3) | 161 (57.5) |
| *3DL1/3DS1/Bw4+* | 68 (25.8) | 38 (26.6) | 30 (24.8) | 66 (27.7) | 74 (26.4) |
| *3DL1/3DS1/Bw4/Bw4+* | 26 (9.8) | 14 (9.8) | 12 (9.9) | 15 (6.3) | 32 (11.4) |
| *2DS2/2DL2/2DL3/C1+* | 79 (29.9) | 44 (30.8) | 35 (28.9) | 82 (34.5) | 80 (28.6) |
| *2DS2/2DL2/2DL3/C1-* | 24 (9.1) | 10 (7.0) | 14 (11.6) | 27 (11.3) | 19 (6.8) |
| *2DS2/2DL2/2DL3/C1C1+* | 34 (12.9) | 22 (15.4) | 12 (9.9) | 35 (14.7) | 29 (10.4) |
| *2DS2/2DL2/2DL3/C1C2+* | 45 (17.0) | 22 (15.4) | 23 (19.0) | 47 (19.7) | 51 (18.2) |
| *2DS2+ /2DL2- /2DL3+ /C1+* | 3 (1.1) | 0 | 3 (2.5) | 1 (0.4) | 3 (1.1) |
| *2DS2+ /2DL2+ /2DL3- /C1+* | 22 (8.3) | 14 (9.8) | 8 (6.6) | 16 (6.7) | 21 (7.5) |
| *2DS2- /2DL2+ /2DL3+ /C1+* | 4 (1.5) | 4 (2.8) | 0 | 4 (1.7) | 3 (1.1) |
| *2DS2- /2DL2- /2DL3+ /C1+* | 99 (37.5) | 52 (36.4) | 47 (38.8) | 84 (35.3) | 116 (41.4) |

MB = multibacillary leprosy (lepromatous leprosy + borderline leprosy); N: number of individuals; n: number of individuals with the KIR genes their HLA ligands.

**^a^**(*P* = 0.03, OR = 1.73, 95% CI = 1.08-2.78, *P* adjusted = 0.014, for ***3DL2/A3/A11+*** in borderline leprosy patients: 36.4% *vs*. 24.8% contacts);

**^b^** (*P* = 0.02, OR = 1.71, 95% CI = 1.08-2.71, for ***3DL2/A3/A11+*** in borderline leprosy patients: 36.4% *vs*. 25.0% healthy subjects);

**^c^**(*P* = 0.005, OR = 0.44, 95% CI = 0.25-0.77, *P* adjusted <0.01 for ***3DL2/A3/A11*+** in lepromatous leprosy patients: 20.28% *vs*. 36.36% borderline leprosy patients);

**^d^** (*P* = 0.03, OR = 0.43, 95% CI = 0.20-0.92, for ***2DS1*/C2-** in lepromatous leprosy patients: 9.1% *vs*. 18.2% borderline leprosy patients)

**^e^** (*P* = 0.04, OR = 2.11, 95% CI = 1.01-4.39, *P* adjusted= 0.058, for ***3DS1*/Bw4-** in borderline leprosy patients: 13.2% *vs*. 6.7% contacts);

**^f^** (*P* = 0.04, , OR = 1.50, 95% CI = 1.02-2.21, for ***2DS5/C2+*** in MB leprosy patients: 29.6% *vs*. 21.8% healthy subjects);

**^g^** (*P* = 0.02, OR = 1.70, 95% CI = 1.08-2.67, *P* adjusted= 0.037, for ***2DS5/C2+*** in lepromatous leprosy patients: 32.17% *vs*. 21.79% healthy subjects);

**^h^** (*P* = 0.02, OR = 3.11, 95% CI = 1.24-7.80, *P* adjusted= 0.027, for ***2DL2/2DL2*/C1/C2+** in lepromatous leprosy patients: 8.4% *vs*. 2.9% healthy subjects);

**^i^** (*P* = 0.02, OR = 2.05, 95% CI = 1.14-3.69, *P* adjusted= 0.014, for ***2DL3/2DL3*/C1/C1+** in borderline leprosy patients: 21.5% *vs*. 11.8% contacts);

**^j^**(*P* = 0.03, , OR = 0.45, 95% CI = 0.21-0.93, *P* adjusted= 0.018, for ***2DS1*/*2DL1*/C2-** in lepromatous leprosy patients: 9.1% *vs*. 18.2% borderline leprosy patients).
