# Supplementary material for: The impact of KIR/HLA genes on the risk of developing multibacillary leprosy
Source: PLoS Negl Trop Dis. 2019 Sep 16;13(9):e0007696. doi: 10.1371/journal.pntd.0007696 (PMC6762192; doi:10.1371/journal.pntd.0007696)
Supplement: S3 Table — (DOCX) [file pntd.0007696.s003.docx]

**Table S3.** *KIR* genotype profiles of multibacillary leprosy patients, their clinical subgroups and controls (healthy household contacts and healthy subjects)

|  | | ***KIR genes*** | | | | | | | | | | | | | | | |  |  |  |  |  |  |  |  |  |  |  |  |  |  |  |  |  |  |  |  |
| --- | --- | --- | --- | --- | --- | --- | --- | --- | --- | --- | --- | --- | --- | --- | --- | --- | --- | --- | --- | --- | --- | --- | --- | --- | --- | --- | --- | --- | --- | --- | --- | --- | --- | --- | --- | --- | --- |
| **Hapl. G** | **Gen.ID** | ***3DL1*** | ***2DL1*** | ***2DL3*** | ***2DS4*** | ***2DL2*** | ***2DL5*** | ***3DS1*** | ***2DS1*** | ***2DS2*** | ***2DS3*** | ***2DS5*** | ***2DL4*** | ***3DL2*** | ***3DL3*** | ***2DP1*** | ***3DP1*** | **MB leprosy**  **N =264**  **n (%)** | **Lepromatous**  **N =143**  **n (%)** | **Borderline**  **N =121**  **n (%)** | | **Contacts**  **N =238**  **n (%)** | **Healthy subjects**  **N =280**  **n (%)** |  |  |  |  |  |  |  |  |  |  |  |  |  |  |
| **AA** | **1** |  |  |  |  |  |  |  |  |  |  |  |  |  |  |  |  | 66 (25.0)**^a^** | 39 (27.3) | 27 (22.3)**^b^** | | 70 (29.4) | 93 (33.2)**^a b^** |  |  |  |  |  |  |  |  |  |  |  |  |  |  |
| **AA** | **195** |  |  |  |  |  |  |  |  |  |  |  |  |  |  |  |  | 1 (0.4) | 1 (0.7) | 0 | | 0 | 0 |  |  |  |  |  |  |  |  |  |  |  |  |  |  |
| **Bx** | **2** |  |  |  |  |  |  |  |  |  |  |  |  |  |  |  |  | 33 (12.5) | 17 (11.9) | 16 (13.2) | | 20 (8.4) | 36 (12.9) |  |  |  |  |  |  |  |  |  |  |  |  |  |  |
| **Bx** | **3** |  |  |  |  |  |  |  |  |  |  |  |  |  |  |  |  | 10 (3.8) | 9 (6.3)**^d^** | 1 (0.8)**^c d^** | | 14 (5.9) **^c^** | 12 (4.3) |  |  |  |  |  |  |  |  |  |  |  |  |  |  |
| **Bx** | **4** |  |  |  |  |  |  |  |  |  |  |  |  |  |  |  |  | 38 (14.4) | 18 (12.6) | 20 (16.5) | | 25 (10.5) | 30 (10.7) |  |  |  |  |  |  |  |  |  |  |  |  |  |  |
| **Bx** | **5** |  |  |  |  |  |  |  |  |  |  |  |  |  |  |  |  | 18 (6.8) | 10 (7.0) | 8 (6.6) | | 21 (8.8) | 22 (7.9) |  |  |  |  |  |  |  |  |  |  |  |  |  |  |
| **Bx** | **6** |  |  |  |  |  |  |  |  |  |  |  |  |  |  |  |  | 13 (4.9) | 7 (4.9) | 6 (5.0) | | 12 (5.0) | 8 (2.9) |  |  |  |  |  |  |  |  |  |  |  |  |  |  |
| **Bx** | **7** |  |  |  |  |  |  |  |  |  |  |  |  |  |  |  |  | 5 (1.9) | 4 (2.8) | 1 (0.8) | | 13 (5.5) | 6 (2.1) |  |  |  |  |  |  |  |  |  |  |  |  |  |  |
| **Bx** | **8** |  |  |  |  |  |  |  |  |  |  |  |  |  |  |  |  | 4 (1.5) | 1 (0.7) | 3 (2.5) | | 2 (0.8) | 5 (1.8) |  |  |  |  |  |  |  |  |  |  |  |  |  |  |
| **Bx** | **9** |  |  |  |  |  |  |  |  |  |  |  |  |  |  |  |  | 6 (2.3) | 2 (1.4) | 4 (3.3) | | 6 (2.5) | 7 (2.5) |  |  |  |  |  |  |  |  |  |  |  |  |  |  |
| **Bx** | **10** |  |  |  |  |  |  |  |  |  |  |  |  |  |  |  |  | 0 | 0 | 0 | | 0 | 1 (0.4) |  |  |  |  |  |  |  |  |  |  |  |  |  |  |
| **Bx** | **11** |  |  |  |  |  |  |  |  |  |  |  |  |  |  |  |  | 3 (1.1) | 1 (0.7) | 2 (1.7) | | 0 | 0 |  |  |  |  |  |  |  |  |  |  |  |  |  |  |
| **Bx** | **12** |  |  |  |  |  |  |  |  |  |  |  |  |  |  |  |  | 1 (0.4) | 0 | 1 (0.8) | | 1 (0.4) | 1 (0.4) |  |  |  |  |  |  |  |  |  |  |  |  |  |  |
| **Bx** | **13** |  |  |  |  |  |  |  |  |  |  |  |  |  |  |  |  | 0 | 0 | 0 | | 2 (0.8) | 1 (0.4) |  |  |  |  |  |  |  |  |  |  |  |  |  |  |
| **Bx** | **15** |  |  |  |  |  |  |  |  |  |  |  |  |  |  |  |  | 0 | 0 | 0 | | 0 | 1 (0.4) |  |  |  |  |  |  |  |  |  |  |  |  |  |  |
| **Bx** | **17** |  |  |  |  |  |  |  |  |  |  |  |  |  |  |  |  | 1 (0.4) | 1 (0.7) | 0 | | 0 | 0 |  |  |  |  |  |  |  |  |  |  |  |  |  |  |
| **Bx** | **18** |  |  |  |  |  |  |  |  |  |  |  |  |  |  |  |  | 0 | 0 | 0 | | 1 (0.4) | 0 |  |  |  |  |  |  |  |  |  |  |  |  |  |  |
| **Bx** | **19** |  |  |  |  |  |  |  |  |  |  |  |  |  |  |  |  | 1 (0.4) | 1 (0.7) | 0 | | 1 (0.4) | 2 (0.7) |  |  |  |  |  |  |  |  |  |  |  |  |  |  |
| **Bx** | **20** |  |  |  |  |  |  |  |  |  |  |  |  |  |  |  |  | 2 (0.8) | 2 (1.4) | 0 | | 2 (0.8) | 1 (0.4) |  |  |  |  |  |  |  |  |  |  |  |  |  |  |
| **Bx** | **21** |  |  |  |  |  |  |  |  |  |  |  |  |  |  |  |  | 1 (0.4) | 0 | 1 (0.8) | | 1 (0.4) | 2 (0.7) |  |  |  |  |  |  |  |  |  |  |  |  |  |  |
| **Bx** | **23** |  |  |  |  |  |  |  |  |  |  |  |  |  |  |  |  | 1 (0.4) | 1 (0.7) | 0 | | 0 | 0 |  |  |  |  |  |  |  |  |  |  |  |  |  |  |
| **Bx** | **24** |  |  |  |  |  |  |  |  |  |  |  |  |  |  |  |  | 0 | 0 | 0 | | 0 | 1 (0.4) |  |  |  |  |  |  |  |  |  |  |  |  |  |  |
| **Bx** | **25** |  |  |  |  |  |  |  |  |  |  |  |  |  |  |  |  | 0 | 0 | 0 | | 1 (0.4) | 0 |  |  |  |  |  |  |  |  |  |  |  |  |  |  |
| **Bx** | **27** |  |  |  |  |  |  |  |  |  |  |  |  |  |  |  |  | 2 (0.8) | 1 (0.7) | 1 (0.8) | | 0 | 1 (0.4) |  |  |  |  |  |  |  |  |  |  |  |  |  |  |
| **Bx** | **28** |  |  |  |  |  |  |  |  |  |  |  |  |  |  |  |  | 3 (1.1) | 0 | 3 (2.5) | | 1 (0.4) | 2 (0.7) |  |  |  |  |  |  |  |  |  |  |  |  |  |  |
| **Bx** | **30** |  |  |  |  |  |  |  |  |  |  |  |  |  |  |  |  | 1 (0.4) | 1 (0.7) | 0 | | 2 (0.8) | 0 |  |  |  |  |  |  |  |  |  |  |  |  |  |  |
| **Bx** | **32** |  |  |  |  |  |  |  |  |  |  |  |  |  |  |  |  | 1 (0.4) | 1 (0.7) | 0 | | 0 | 0 |  |  |  |  |  |  |  |  |  |  |  |  |  |  |
| **Bx** | **36** |  |  |  |  |  |  |  |  |  |  |  |  |  |  |  |  | 1 (0.4) | 0 | 1 (0.8) | | 0 | 0 |  |  |  |  |  |  |  |  |  |  |  |  |  |  |
| **Bx** | **43** |  |  |  |  |  |  |  |  |  |  |  |  |  |  |  |  | 0 | 0 | 0 | | 1 (0.4) | 0 |  |  |  |  |  |  |  |  |  |  |  |  |  |  |
| **Bx** | **44** |  |  |  |  |  |  |  |  |  |  |  |  |  |  |  |  | 0 | 0 | 0 | | 1 (0.4) | 0 |  |  |  |  |  |  |  |  |  |  |  |  |  |  |
| **Bx** | **51** |  |  |  |  |  |  |  |  |  |  |  |  |  |  |  |  | 0 | 0 | 0 | | 1 (0.4) | 0 |  |  |  |  |  |  |  |  |  |  |  |  |  |  |
| **Bx** | **62** |  |  |  |  |  |  |  |  |  |  |  |  |  |  |  |  | 0 | 0 | 0 | | 1 (0.4) | 0 |  |  |  |  |  |  |  |  |  |  |  |  |  |  |
| **Bx** | **64** |  |  |  |  |  |  |  |  |  |  |  |  |  |  |  |  | 1 (0.4) | 1 (0.7) | 0 | | 0 | 1 (0.4) |  |  |  |  |  |  |  |  |  |  |  |  |  |  |
| **Bx** | **68** |  |  |  |  |  |  |  |  |  |  |  |  |  |  |  |  | 1 (0.4) | 1 (0.7) | 0 | | 3 (1.3) | 3 (1.1) |  |  |  |  |  |  |  |  |  |  |  |  |  |  |
| **Bx** | **69** |  |  |  |  |  |  |  |  |  |  |  |  |  |  |  |  | 10 (3.8) | 4 (2.8) | 6 (5.0) | | 5 (2.1) | 4 (1.4) |  |  |  |  |  |  |  |  |  |  |  |  |  |  |
| **Bx** | **70** |  |  |  |  |  |  |  |  |  |  |  |  |  |  |  |  | 4 (1.5) | 2 (1.4) | 2 (1.7) | | 4 (1.7) | 5 (1.8) |  |  |  |  |  |  |  |  |  |  |  |  |  |  |
| **Bx** | **71** |  |  |  |  |  |  |  |  |  |  |  |  |  |  |  |  | 6 (2.3) | 3 (2.1) | 3 (2.5) | | 2 (0.8) | 6 (2.1) |  |  |  |  |  |  |  |  |  |  |  |  |  |  |
| **Bx** | **72** |  |  |  |  |  |  |  |  |  |  |  |  |  |  |  |  | 4 (1.5) | 4 (2.8) | 0 | | 3 (1.3) | 5 (1.8) |  |  |  |  |  |  |  |  |  |  |  |  |  |  |
| **Bx** | **73** |  |  |  |  |  |  |  |  |  |  |  |  |  |  |  |  | 3 (1.1) | 2 (1.4) | 1 (0.8) | | 4 (1.7) | 5 (1.8) |  |  |  |  |  |  |  |  |  |  |  |  |  |  |
| **Bx** | **74** |  |  |  |  |  |  |  |  |  |  |  |  |  |  |  |  | 0 | 0 | 0 | | 1 (0.4) | 0 |  |  |  |  |  |  |  |  |  |  |  |  |  |  |
| **Bx** | **76** |  |  |  |  |  |  |  |  |  |  |  |  |  |  |  |  | 3 (1.1) | 2 (1.4) | 1 (0.8) | | 0 | 3 (1.1) |  |  |  |  |  |  |  |  |  |  |  |  |  |  |
| **Bx** | **79** |  |  |  |  |  |  |  |  |  |  |  |  |  |  |  |  | 2 (0.8) | 0 | 2 (1.7) | | 1 (0.4) | 0 |  |  |  |  |  |  |  |  |  |  |  |  |  |  |
| **Bx** | **81** |  |  |  |  |  |  |  |  |  |  |  |  |  |  |  |  | 3 (1.1) | 2 (1.4) | 1 (0.8) | | 1 (0.4) | 2 (0.7) |  |  |  |  |  |  |  |  |  |  |  |  |  |  |
| **Bx** | **88** |  |  |  |  |  |  |  |  |  |  |  |  |  |  |  |  | 1 (0.4) | 0 | 1 (0.8) | | 0 | 0 |  |  |  |  |  |  |  |  |  |  |  |  |  |  |
| **Bx** | **90** |  |  |  |  |  |  |  |  |  |  |  |  |  |  |  |  | 2 (0.8) | 0 | 2 (1.7) | | 3 (1.3) | 6 (2.1) |  |  |  |  |  |  |  |  |  |  |  |  |  |  |
| **Bx** | **91** |  |  |  |  |  |  |  |  |  |  |  |  |  |  |  |  | 0 | 0 | 0 | | 0 | 1 (0.4) |  |  |  |  |  |  |  |  |  |  |  |  |  |  |
| **Bx** | **93** |  |  |  |  |  |  |  |  |  |  |  |  |  |  |  |  | 0 | 0 | 0 | | 1 (0.4) | 0 |  |  |  |  |  |  |  |  |  |  |  |  |  |  |
| **Bx** | **94** |  |  |  |  |  |  |  |  |  |  |  |  |  |  |  |  | 1 (0.4) | 1 (0.7) | 0 | | 0 | 1 (0.4) |  |  |  |  |  |  |  |  |  |  |  |  |  |  |
| **Bx** | **106** |  |  |  |  |  |  |  |  |  |  |  |  |  |  |  |  | 0 | 0 | 0 | | 1 (0.4) | 0 |  |  |  |  |  |  |  |  |  |  |  |  |  |  |
| **Bx** | **112** |  |  |  |  |  |  |  |  |  |  |  |  |  |  |  |  | 1 (0.4) | 1 (0.7) | 0 | | 2 (0.8) | 0 |  |  |  |  |  |  |  |  |  |  |  |  |  |  |
| **Bx** | **117** |  |  |  |  |  |  |  |  |  |  |  |  |  |  |  |  | 0 | 0 | 0 | | 0 | 1 (0.4) |  |  |  |  |  |  |  |  |  |  |  |  |  |  |
| **Bx** | **118** |  |  |  |  |  |  |  |  |  |  |  |  |  |  |  |  | 1 (0.4) | 0 | 1 (0.8) | | 0 | 0 |  |  |  |  |  |  |  |  |  |  |  |  |  |  |
| **Bx** | **159** |  |  |  |  |  |  |  |  |  |  |  |  |  |  |  |  | 1 (0.4) | 0 | 1 (0.8) | | 0 | 0 |  |  |  |  |  |  |  |  |  |  |  |  |  |  |
| **Bx** | **166** |  |  |  |  |  |  |  |  |  |  |  |  |  |  |  |  | 0 | 0 | 0 | | 0 | 1 (0.4) |  |  |  |  |  |  |  |  |  |  |  |  |  |  |
| **Bx** | **188** |  |  |  |  |  |  |  |  |  |  |  |  |  |  |  |  | 0 | 0 | 0 | | 0 | 1 (0.4) |  |  |  |  |  |  |  |  |  |  |  |  |  |  |
| **Bx** | **202** |  |  |  |  |  |  |  |  |  |  |  |  |  |  |  |  | 1 (0.4) | 0 | 1 (0.8) | | 1 (0.4) | 0 |  |  |  |  |  |  |  |  |  |  |  |  |  |  |
| **Bx** | **242** |  |  |  |  |  |  |  |  |  |  |  |  |  |  |  |  | 0 | 0 | 0 | | 0 | 1 (0.4) |  |  |  |  |  |  |  |  |  |  |  |  |  |  |
| **Bx** | **244** |  |  |  |  |  |  |  |  |  |  |  |  |  |  |  |  | 1 (0.4) | 1 (0.7) | 0 | | 0 | 0 |  |  |  |  |  |  |  |  |  |  |  |  |  |  |
| **Bx** | **260** |  |  |  |  |  |  |  |  |  |  |  |  |  |  |  |  | 1 (0.4) | 0 | 1 (0.8) | | 0 | 0 |  |  |  |  |  |  |  |  |  |  |  |  |  |  |
| **Bx** | **293** |  |  |  |  |  |  |  |  |  |  |  |  |  |  |  |  | 1 (0.4) | 0 | 1 (0.8) | | 0 | 0 |  |  |  |  |  |  |  |  |  |  |  |  |  |  |
| **Bx** | **313** |  |  |  |  |  |  |  |  |  |  |  |  |  |  |  |  | 0 | 0 | 0 | | 1 (0.4) | 0 |  |  |  |  |  |  |  |  |  |  |  |  |  |  |
| **Bx** | **324** |  |  |  |  |  |  |  |  |  |  |  |  |  |  |  |  | 0 | 0 | 0 | | 0 | 1 (0.4) |  |  |  |  |  |  |  |  |  |  |  |  |  |  |
| **Bx** | **325** |  |  |  |  |  |  |  |  |  |  |  |  |  |  |  |  | 1 (0.4) | 1 (0.7) | 0 | | 0 | 1 (0.4) |  |  |  |  |  |  |  |  |  |  |  |  |  |  |
| **Bx** | **363** |  |  |  |  |  |  |  |  |  |  |  |  |  |  |  |  | 1 (0.4) | 0 | 1 (0.8) | | 0 | 0 |  |  |  |  |  |  |  |  |  |  |  |  |  |  |
| **Bx** | **382** |  |  |  |  |  |  |  |  |  |  |  |  |  |  |  |  | 1 (0.4) | 0 | 1 (0.8) | | 1 (0.4) | 0 |  |  |  |  |  |  |  |  |  |  |  |  |  |  |
| **Bx** | **392** |  |  |  |  |  |  |  |  |  |  |  |  |  |  |  |  | 0 | 0 | 0 | | 1 (0.4) | 0 |  |  |  |  |  |  |  |  |  |  |  |  |  |  |
| **Bx** | **NAS0** |  |  |  |  |  |  |  |  |  |  |  |  |  |  |  |  | 1 (0.4) | 1 (0.7) | 0 | | 1 (0.4) | 0 |  |  |  |  |  |  |  |  |  |  |  |  |  |  |
| **Bx** | **NAS1** |  |  |  |  |  |  |  |  |  |  |  |  |  |  |  |  | 0 | 0 | 0 | | 2 (0.8) | 0 |  |  |  |  |  |  |  |  |  |  |  |  |  |  |
|  | **Number of *KIR* genotypes** | | | | | | | | | | | | | | | | | 46 | 32 | 31 | | 42 | 37 |  |  |  |  |  |  |  |  |  |  |  |  |  | 48 |

Hapl. G: Haplotype group; Gen. ID: Genotype ID assigned by the Allele Frequencies Net Database (April 2018); NAS: Not yet Assigned ID; black box = gene detected; white box = gene absent, no detected; MB = multibacillary leprosy (lepromatous leprosy + borderline leprosy); Ind. H: Individuals healthy; N: number of individuals; n: number of individuals with genotype ID.

**^a^** (*P* = 0.044, OR = 0.67, 95% CI = 0.46-0.97, for **Genotype ID 1** in MB leprosy patients: 25.0% *vs*. 33.2% healthy subjects);

**^b^** (*P* = 0.038, OR = 0.57, 95% CI = 0.35-0.94, for **Genotype ID 1** in borderline leprosy patients: 22.3% *vs*. 33.2% healthy subjects);

**^c^** (*P* = 0.047, OR = 0.13, 95% CI = 0.003-0.89, for **Genotype ID 3** in borderline leprosy patients: 0.8% *vs*. 5.9% contacts);

**^d^** (*P* = 0.046, OR = 8.06, 95% CI = 1.08-undefined, for **Genotype ID 3** in lepromatous leprosy patients: 6.3% *vs*. 0.8% borderline leprosy patients);
